# Pattern of distant metastases in colorectal cancer: a SEER based study

**DOI:** 10.18632/oncotarget.6130

**Published:** 2015-10-15

**Authors:** Miaozhen Qiu, Jianming Hu, Dajun Yang, David Peter Cosgrove, Ruihua Xu

**Affiliations:** ^1^ Department of Medical Oncology, Sun Yat-Sen University Cancer Center, State Key Laboratory of Oncology in South China, Collaborative Innovation Center for Cancer Medicine, Guangzhou, China; ^2^ Department of Oncology, The Sidney Kimmel Comprehensive Cancer Center, The Johns Hopkins University School of Medicine, Baltimore, MD, USA; ^3^ Department of Medical Imaging Department, Sun Yat-Sen University Cancer Center, State Key Laboratory of Oncology in South China, Collaborative Innovation Center for Cancer Medicine, Guangzhou, China; ^4^ Department of Experimental Research, Sun Yat-Sen University Cancer Center, State Key Laboratory of Oncology in South China, Collaborative Innovation Center for Cancer Medicine, Guangzhou, China

**Keywords:** incidence, metastases, colon cancer, rectal cancer, SEER

## Abstract

More and more evidences suggest that primary colon and rectum tumors should not be considered as a single disease entity. In this manuscript, we evaluate the metastatic patterns of colon and rectum cancers and analyze the potential distribution of metastatic disease in these two malignancies. Data queried for this analysis include colorectal adenocarcinoma (2010-2011) from the Surveillance, Epidemiology, and End Results Program (SEER) database. Metastatic distribution information was provided for liver, lung, bone and brain. All of statistical analyses were performed using the Intercooled Stata 13.0 (Stata Corporation, College Station, TX). All statistical tests were two-sided. Totally, there were 46,027 eligible patients for analysis. We found that colon cancer had a higher incident rate of liver metastasis than rectum cancer (13.8% vs 12.3%), while rectum cancer had a higher incident rate of lung (5.6% vs 3.7%) and bone (1.2% vs 0.8%) metastasis than colon cancer, P<0.001. Colorectal cancer patients with lung metastasis had a higher risk of bone (10.0% vs 4.5%) or brain metastasis (3.1% vs 0.1%) than patients without lung metastases. The 1-year cause-specific survival was not significant different for bone or brain metastasis patients with and without lung metastasis (32.9% vs 38.7%, *P*=0.3834 for bone, 25.8% vs 36.9%, *P*=0.6819 for brain). Knowledge of these differences in metastatic patterns may help to better guide pre-treatment evaluation of colorectal cancer patients, especially in making determinations regarding curative-intent interventions.

## INTRODUCTION

Colorectal cancer (CRC) is the third most commonly diagnosed cancer among men and women in the United States [1, 2]. Though colon and rectum cancer are often referred to as colorectal cancer, they are actually distinct disease entities. They have a different inherent prognosis, different potential for treatment response (and indeed different standard treatment options) and may have different metastatic patterns [3]. Previous studies had generated insight into metastatic patterns and showed that different primary cancers tended to metastasize with different frequencies and to different sites [4, 5]. At the time of diagnosis, about 20% of CRC patients have already developed metastatic diseases [6]. It is well known that the most common metastatic site for CRC patients is liver, followed by lung [4, 7, 8]. Several clinical studies regarding CRC have already demonstrated that there are differences in metastatic patterns between colon and rectum cancer. A nationwide retrospective review of 5,817 pathological records of CRC patients showed that rectum cancer patients more often had metastasis at extra-abdominal sites while patients with colon cancer had a higher rate of abdominal metastasis [5].

Review of autopsy data from patients who died from colorectal cancer shows that liver is the only site of metastatic disease in one third of patients. Studies of selected CRC patients undergoing surgery to remove liver metastases have shown that cure is certainly attainable in this population [9]. Therefore patients diagnosed with potentially resectable metastatic CRC should ideally undergo an upfront evaluation by a multidisciplinary team to maximize the curative potential [10-12]. Thus it is crucial to exclude extrahepatic (or extra-pulmonary) metastasis before local treatment. A clear understanding of the metastatic pattern and distribution becomes especially important.

In this Surveillance, Epidemiology and End Results (SEER)-based study, we compare the metastatic pattern of colon and rectum cancers. Moreover, we analyze the distribution of metastatic site(s) for these patients.

## RESULTS

### Patient characteristics

The study group consisted of 46,027 patients, including 24,135 men (52.4%) and 21,892 women (47.6%). Colon cancer was found in 35,882 (77.9%) cases, compared with 10,145 rectal cancer cases (22.1%). The incidence rate of colon cancer was 3.5 times greater than the rectal cancer in the current cohort (Table 1). The median age was 67 for colon cancer patients and 62 for rectum cancer patients.

**Table 1:** Clinical features and metastasis sites

Features	Liver metastasis (%)		*P* value*	Lung metastasis (%)		*P* value#	Bone metastasis (%)		*P* value&	Brain metastasis (%)		*P* value$
	No	Yes		No	Yes		No	Yes		No	Yes	
Sex												
Women	18,802 (87.7)	2,628 (12.3)	<0.001	20,542 (96.1)	823 (3.9)	0.005	21,190 (99.3)	160 (0.7)	0.032	21,282 (99.7)	54 (0.3)	0.487
Men	20,162 (85.4)	3,452 (14.6)		22,492 (95.6)	1,029 (4.4)		23,291 (99.1)	220 (0.9)		23,456 (99.8)	52 (0.2)	
Age			<0.001			<0.001			<0.001			<0.001
Mean	66.00	68.91		65.64	68.56		65.58	61.96		65.55	64.24	
SD	13.78	15.47		13.81	15.25		13.79	13.14		13.79	13.07.	
Median	66	62		66	63		66	62		66	64	
Ethnicity			<0.001			<0.001			0.057			0.037
Caucasian	30,615 (87.1)	4,526 (12.9)		33,652 (96.1)	1,358 (3.9)		34,720 (99.2)	281 (0.8)		34,900 (99.8)	81 (0.2)	
African American	4,362 (82.1)	952 (17.9)		5,015 (94.6)	287 (5.4)		5,237 (98.8)	63 (1.2)		5,288 (99.7)	17 (0.3)	
Asian	3,166 (86.7)	487 (13.3)		3,475 (95.3)	171 (4.7)		3,604 (99.3)	27 (0.7)		3,624 (99.9)	5 (0.1)	
Other	544 (84.1)	103 (15.9)		607 (95.0)	32 (5.0)		634 (98.9)	7 (1.1)		638 (99.5)	3 (0.5)	
Unknown	277 (95.8)	12 (4.2)		285 (98.6)	4 (1.4)		286 (99.3)	2 (0.7)		400 (99.8)	1 (0.2)	
Primary tumor sites			<0.001			<0.001			<0.001			0.060
Right colon	16,009 (87.5)	2,284 (12.5)		17,672 (96.9)	562 (3.1)		18,108 (99.3)	120 (0.7)		18,167 (99.7)	55 (0.3)	
Left colon	14307 (84.7)	2581 (15.3)		16,095 (95.6)	741 (4.4)		16,673 (99.1)	146 (0.9)		16,782 (99.8)	33 (0.2)	
Rectal	8,648 (87.7)	1,215 (12.3)		9,267 (94.4)	549 (5.6)		9,700 (98.8)	114 (1.2)		9,789 (99.8)	18 (0.2)	
Grade			<0.001			<0.001			<0.001			<0.001
Well	3,518 (93.3)	251 (0.7)		3,679 (97.9)	79 (2.1)		3,747 (99.6)	14 (0.4)		3,759 (99.9)	2 (0.1)	
Moderate	25,867 (87.9)	3,545 (12.1)		28,268 (96.3)	1,071 (3.7)		29,123 (99.4)	180 (0.6)		29,236 (99.8)	59 (0.2)	
Poorly	5,383 (83.3)	1,076 (16.7)		6,154 (95.7)	274 (4.3)		6,357 (98.8)	74 (1.2)		6,395 (99.6)	28 (0.4)	
Undifferentiated	828 (84.4)	153 (15.6)		946 (96.5)	34 (3.5)		966 (98.6)	14 (1.4)		978 (99.9)	1 (0.1)	
Unknown	3,368 (76.1)	1,055 (23.9)		3,987 (91.0)	394 (9.0)		4,288 (97.8)	98 (2.2)		4,370 (99.6)	16 (0.4)	
Insurance			<0.001			<0.001			0.044			0.237
Insured	36,682 (86.8)	5,595 (13.2)		40,430 (96.0)	1,701 (4.0)		41,752 (99.2)	352 (0.8)		41,991 (99.8)	98 (0.2)	
Uninsured	1,393 (79.9)	350 (20.1)		1,617 (93.1)	120 (6.9)		1,718 (98.7)	23 (1.3)		1,732 (99.6)	7 (0.4)	
Unknown	889 (86.8)	135 (13.2)		987 (97.0)	31 (3.0)		1,011 (99.5)	5 (0.5)		1,015 (99.9)	1 (0.1)	
Marital status			<0.001			<0.001			0.465			0.084
Married	20,781 (87.0)	3,116 (13.0)		22,945 (96.3)	876 (3.7)		23,619 (99.2)	192 (0.8)		23,736 (99.8)	58 (0.2)	
Unmarried	15,769 (85.6)	2,650 (14.4)		17,477 (95.2)	878 (4.8)		18,169 (99.1)	167 (0.9)		18,290 (99.7)	47 (0.3)	
Unknown	2,414 (88.5)	314 (11.5)		2,612 (96.4)	98 (3.6)		2,693 (99.2)	21 (0.8)		2,712 (99.9)	1 (0.1)	

SD: Standard deviation.

* The comparison between patients with and without liver metastasis.

# The comparison between patients with and without lung metastasis.

& The comparison between patients with and without bone metastasis.

$ The comparison between patients with and without brain metastasis.

### Metastasis pattern

At the time of diagnosis, stage IV disease accounted for 18.1% (8,347/46,027) of all the CRC cases. The database only had metastatic information related to liver, lung, bone and brain metastasis. Patients who had metastasis to either one of the four sites accounted for 92.9% (7,759/8,347) of stage IV diseases. Clinical features of metastatic CRC patients were presented in Table 1.

Though liver was the most common metastatic site for both colon and rectum cancer, colon cancer had a higher incident rate of liver metastasis (13.8% *vs* 12.3%, *P* < 0.001) than rectal cancer, while rectum cancer had a higher incidence rate of lung (5.6% *vs* 3.7%, *P* < 0.001) and bone metastasis (1.2% *vs* 0.8%, *P* < 0.001) than colon cancer. We found that left colon had a higher metastatic rate than right colon, specifically to the liver (15.3% *vs* 12.5%), lung (4.4% *vs* 3.1%) and bone (0.9% *vs* 0.7%).

As for gender, men had a higher risk than women in terms of liver, lung and bone metastasis, but there was no difference for brain metastasis between the genders (Table 1).

We found that African Americans had a higher incident rate of liver (17.9%) and lung (5.4%) metastases than Caucasian, Asian and other race counterparts. Uninsured patients had more metastasis to liver, lung and bone than insured patients. Moreover, unmarried patients also had more metastasis to liver and lung than married patients.

### Combination of metastases

Many patients developed more than one site of metastatic diseases. Table 2 summarized all the possible combinations of these four sites of metastasis. About 10.01% of colon cancer and 7.66% of rectum cancer patients had only liver metastasis. Rare CRC patients had only brain or bone metastasis at the time of diagnosis. The most common two-site metastasis combination was liver and lung (2.24% for colon cancer and 2.84% for rectal cancer). We also found that rectum cancer was more likely to have two-site metastasis than colon cancer, especially for lung and liver, lung and bone as well as liver and bone combination.

**Table 2:** Frequencies of combination metastasis

Features	Colon cancer		Rectal cancer		*P* value
	Number	(%)	Number	(%)	
One site					
Only liver	3,590	10.01	777	7.66	<0.001
Only lung	283	0.79	172	1.70	<0.001
Only bone	33	0.09	27	0.27	<0.001
Only brain	22	0.06	6	0.06	0.938
Two sites					
Lung and liver	803	2.24	288	2.84	<0.001
Lung and bone	22	0.06	15	0.15	0.007
Lung and brain	8	0.02	4	0.04	0.345
Liver and bone	88	0.25	33	0.33	0.165
Liver and brain	9	0.03	2	0.02	0.760
Bone and brain	1	0.003	1	0.007	0.340
Three sites					
Lung and liver and bone	90	0.25	28	0.28	0.658
Lung and liver and brain	23	0.06	1	0.03	0.035
Liver and bone and brain	5	0.01	0	0	--
Bone and brain and lung	2	0.005	0	0	--
Four sites					
Liver and lung and bone and brain	8	0.02	3	0.03	0.676

**Figure 1:**
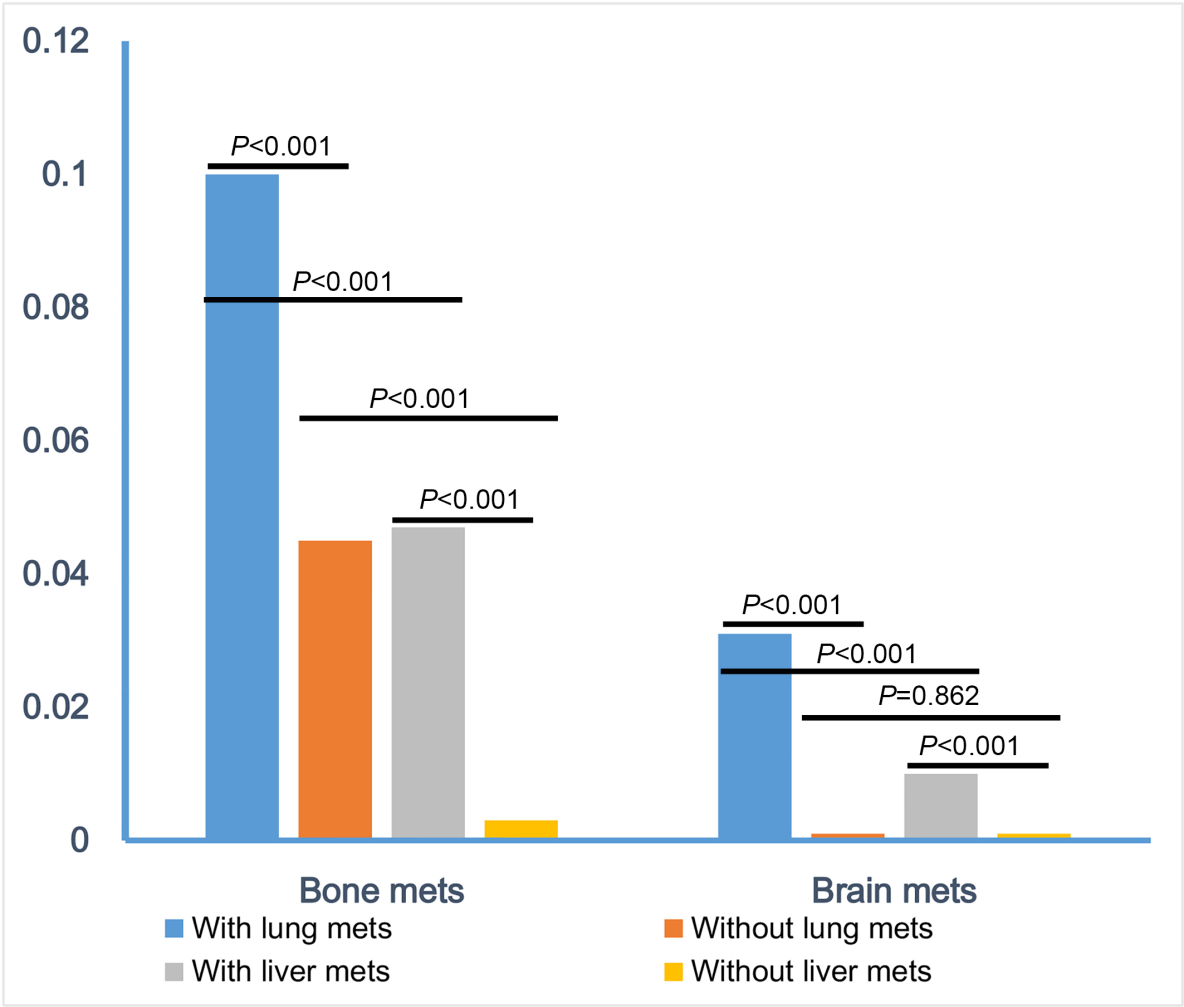
Comparisons of combination-metastatic rate

To know more about the interaction among these sites of metastasis, we compared the risk of bone and brain metastasis between patients with and without lung or liver metastasis. We found that patients with lung metastasis had a higher risk of bone (10.0% *vs* 4.5%, *P* < 0.001) or brain metastasis (3.1% *vs* 0.1%, *P* < 0.001) than patients without (Figure 1). Though a similar phenomenon was noted for liver metastasis, with higher risk of bone (4.7% *vs* 0.3%, *P* < 0.001) and brain metastasis (1.0% *vs* 0.1%, *P* < 0.001) for liver metastasis patients than those without, CRC patients with lung metastasis had a higher incidence rate of bone or brain metastasis than patients with liver metastasis.

### Survival

**Figure 2:**
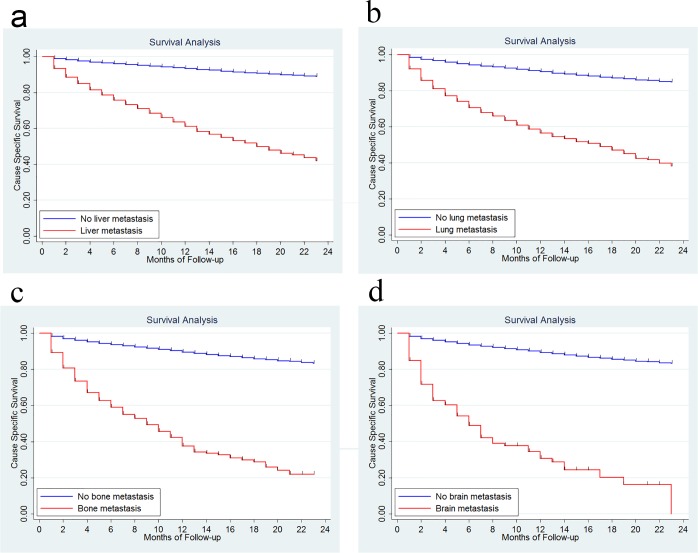
Survival analysis in patients with and without liver metastasis **2a.**, with and without lung metastasis **2b.**, with and without bone metastasis **2c.**, with and without brain metastasis **2d.**

**Figure 3:**
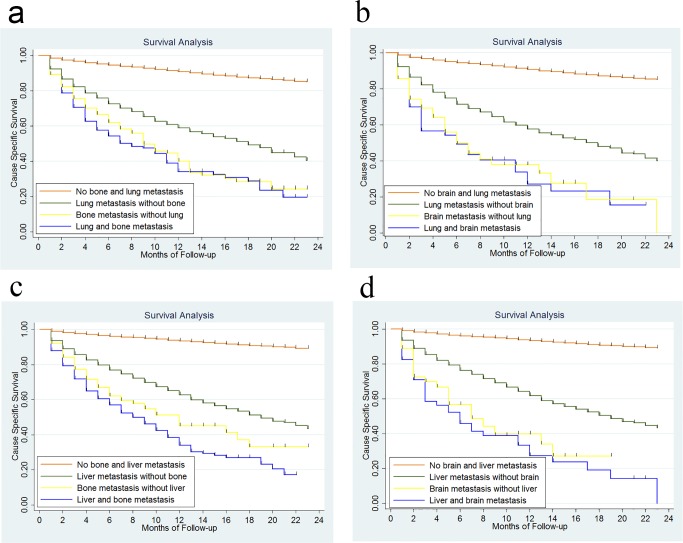
Survival analysis according to metastasis to bone and lung **3a.**, metastasis to brain and lung **3b.**, metastasis to bone and liver **3c.**, metastasis to brain and liver **3d.**.

In this study, 4859 deaths (10.56 %) were observed. Since the time of follow-up is short, we only calculated the 1-year cause-specific survival (CSS). It was 88.4% for the whole group patients. The 1-year CSS was 60.2% *vs* 93.1% for patients with and without liver metastasis, 55.5% *vs* 90.2% for patients with and without lung metastasis, 36.2% *vs* 89.3% for patients with and without bone metastasis and 29.6% *vs* 90.0% for patients with and without brain metastasis (Figure 2). Patients with both bone and lung metastasis had a slightly worse 1-year CSS than those with only bone metastasis, 32.9% *vs* 38.7%, but the difference was not significant, *P* = 0.3834. Similarly, the 1-year CSS was not significant different for brain metastasis patients with and without lung metastasis, 25.8% *vs* 36.9%, *P* = 0.6819. There was no significant 1-year CSS difference for bone metastasis patients with and without liver metastasis, 32.8% *vs* 43.7, *P* = 0.0695, or for brain metastasis patients with and without liver metastasis, 26.4% *vs* 38.3%, *P* = 0.4238 (Figure 3).

## DISCUSSION

In the present study, we clarified the following two points: (1) confirming major differences in metastatic frequencies between colon and rectum cancer; (2) identifying risks of specific combinations of metastatic sites in CRC. These differences may have significant implications for clinical decision-making.

As for the metastatic patterns of colon and rectum cancers, we reaffirm that both colon and rectum cancer predominantly metastasize to liver. Moreover, colon cancer patients presented with liver metastasis more often, whereas rectum cancer patients presented with lung and bone metastasis with relatively higher frequency. This was consistent with previous reports that colon cancer patients present with abdominal metastasis more often, while rectum cancer patients present more frequently with extra-abdominal metastatic sites [5]. Although brain is an extra-abdominal organ, there is no difference in the rate of brain metastasis between colon cancer and rectum cancer in our study, while other report showed a higher risk of brain metastasis in patients with primary rectum cancer [5]. Previous studies have reported that the incidence rate of brain metastasis in CRC patients ranges from 1-3% [13, 14]. However, metastasis to brain is most commonly a late-stage phenomenon [15, 16], whereas our study only assessed patients with synchronous metastasis. The rate of brain metastasis is extremely low at the time of diagnosis, only 0.2% in this dataset.

Moreover, we found that left colon cancer patients had higher incident rate of metastasis to liver, lung as well as bone than right colon cancer patients. More and more studies have indicated that right- and left-sided colon cancers should not be regarded as a single entity. They are significantly different regarding epidemiological, clinical and histological parameters [17–19]. One study by Benedix et al. reported 17,641 patients with colon cancer and found that hepatic and pulmonary metastasis were more frequently found in those with left-sided colon cancer. This is consistent with our data [20].

We also found that African-American patients had a higher risk of lung and liver metastasis. Phatak UR et al. demonstrated that a greater proportion of African American patients presented with metastatic disease than Caucasian or Asian people [21], but they didn't mention the detailed metastatic sites. Of note, we found that uninsured patients had more metastasis to liver, lung and bone than insured patients. Unmarried patients had more metastasis to liver and lung than married patients. Previous studies showed that spouse might provide social support and encourage the patients to seek medical treatment [22, 23].

Knowledge of differences in metastatic patterns may be useful in making diagnostic and treatment decisions. Since liver and lung metastasis are most common, current guidelines suggest that regular imaging of these sites should be maintained, such as computed tomography (CT) or Magnetic Resonance Imaging (MRI). However, in the case of unusual lesions or with specific symptoms, imaging of other sites should be employed, especially for rectum cancer patients who have a higher risk of extra-abdominal metastasis or for left-side colon and Africa American patients who have a higher overall risk of metastasis.

There are few reports about the combination of metastasis in CRC patients. In Hugen's study, they analyzed different metastatic patterns among different histological types and found that adenocarcinoma patients exhibited the highest percentage of liver metastasis compared with mucinous adenocarcinoma and signet-ring cell carcinoma [5]. We found that around 10% of colon cancer patients and 7% of rectum cancer patients would have liver only metastasis at the time of diagnosis. Very few patients had only brain or bone metastasis. Therefore, in the scenario of a primary colon cancer and a bone metastasis, we should focus on the potential for additional metastatic deposits. Furthermore, we found that patients with lung metastasis have a higher risk of bone and brain metastasis than those without lung metastasis, or those with liver metastasis. Primary lung cancer frequently metastasizes to bone as well as brain [24–27], so this pattern of spread has precedent. The internal mechanism of these distribution patterns remains unknown. Some studies have suggested that there is cross-talk among lung tumor cells, the bone microenvironment and the immune system, which lead to bone metastasis formation in primary non-small cell lung cancer patients [28]. This important finding should be helpful for us to screen this subgroup of patients. For CRC patients who already have lung or liver metastasis, we should also pay more attention to the potential of bone and brain metastasis. This is extremely important when making determinations regarding curative-intent interventions.

To our knowledge, this is the first SEER based study focusing solely on the metastatic pattern of colon cancer and rectum cancer, considering them separate entities. However, there are obvious limitations due to the retrospective nature of this study, as outlined below. First of all, it is important to note that the database only provides data between 2010 and 2011. Furthermore, we only have information on synchronous metastasis to liver, lung, bone and brain, a relative minority compared to those patients who will develop metachronous lesions. These limitations may have led to an underestimation of other sites of metastasis, but as we have noted, the four sites of metastasis assessed account for 90% of stage IV colorectal cancer patients. Moreover, we also realize that this bias potentially applies to both colon and rectal cancer. Overall, this is the first review to confirm the strong potential for bone and brain metastasis in patients who already have lung metastasis. Based on the differences of metastatic patterns in colon and rectum cancer, we suggest that clinicians take the primary tumor site into account when designing diagnostic and treatment algorithms.

In conclusion, based on this SEER data, we found that colon cancer patients present with liver metastasis more often, whereas rectum cancer patients present relatively more frequently with lung and bone metastasis. Males or African American patients were more likely to develop metastasis to lung, liver, as well as bone. Patients with known lung metastasis have a higher risk of bone and brain metastasis.

## MATERIALS AND METHODS

### Database

The SEER program is the largest publicly available cancer dataset. It is a population-based cancer registry covering approximately 26.4% of the US population across several disparate geographic regions.

We hypothesize that SEER is a good database from which to analyze the distant metastasis pattern for colon and rectum cancers. However SEER does not currently include any information on location of metastases in the standard research data. We wrote letters to the SEER service center and were told that as part of collaborative stage, they did recently begin to collect this metastases information for cases diagnosed after 2004+, but they do not release it as part of their standard data. In order to pursue the data, we sent an application through a SEER custom data request and received their permission. This database only includes metastasis to the bone, brain, liver and lung at the time of diagnosis. Moreover, though liver metastasis and other metastatic sites are initially planned for 2004+ cases, there are actually only trackable data for 2010+ cases. Using this dataset, we can only analyze the metastasis at the time of diagnosis, which means that metachronous metastasis is not within the scope of this manuscript.

The dataset we used for analysis was “Incidence-SEER 18 Custom Data (with CS mets at DX fields), Nov 2013 Sub (2010-2011) < Katrina/Rita Population Adjustment>”.

### Outcome variables

The anatomic sub-sites of the left colon, right colon and rectum were categorized according to the International Classification of Diseases for Oncology, third edition (ICD-0-3) topography codes. Right-sided colon cancers were identified with the following SEER cancer site codes: cecum (ICD-0-3 code C18.0), ascending colon (Code C18.2), hepatic flexure (Code C18.3) and transverse colon (Code C18.4). Left-sided colon cancers were identified with codes: splenic flexure (Code C18.5), descending colon (code C18.6), sigmoid colon (code C18.7) and rectosigmoid (code C19.9). Rectal cancer was identified as code C20.9.

For the purposes of this manuscript, only the adenocarcinoma pathologic type (SEER histology codes 8140 to 8147, 8210 to 8211, 8220 to 8221, 8260 to 8263, 8480 to 8481 and 8490) is included. We excluded cases without follow-up records and those who had multiple tumors and CRC was not the first tumor.

According to the Collaborative stage data collection system, user documentation and coding instructions, the output of metastasis to lung, bone, brain and liver will present with 4 different codes. 0 means no metastasis to the site; 1 means confirmed metastasis to the site; 8 means not applicable; and 9 means whether this site is involved is unknown and there is no supportive documentation in patient record. We focused on the code 0 and code 1.

### Statistical analysis

The patients’ demographic and tumor characteristics were summarized with descriptive statistics. Comparisons of categorical variables among different groups of patients were performed using the Chi square test, and continuous variables were compared using Student's t test. CSS was calculated from the date of diagnosis to the date of cancer specific death. Deaths attributed to CRC were treated as events and deaths from other causes were treated as censored observations. Survival function estimation and comparison among different variables were performed using Kaplan-Meier estimates and the log-rank test. All of statistical analyses were performed using the Intercooled Stata 13.0 (Stata Corporation, College Station, TX). Statistical significance was set at two-sided *P* < 0.05. This study was deemed exempt from institutional review board approval by Sun Yat-sen University Cancer Center and informed consent was waived.
